# Dermatofibrosarcoma Protuberans of the Clavicular Skin in a 70-Year-Old Woman: Case Report and Management

**DOI:** 10.7759/cureus.97887

**Published:** 2025-11-26

**Authors:** Dorothy S Peng, Amy Shen, Yuna Kang, Rebecca K Yamamoto, Paul Levins

**Affiliations:** 1 Dermatology, Department of Medicine, University of California Los Angeles David Geffen School of Medicine, Los Angeles, USA; 2 Pathology and Laboratory Medicine, University of California Los Angeles David Geffen School of Medicine, Los Angeles, USA; 3 Department of Medicine, John A. Burns School of Medicine, University of Hawai‘i, Honolulu, USA

**Keywords:** cd34 immunohistochemistry, col1a1 rearrangement, cutaneous sarcoma, dermatofibrosarcoma protuberans, mohs micrographic surgery (mms), soft-tissue sarcoma

## Abstract

Dermatofibrosarcoma protuberans (DFSP) is a rare, low-grade soft tissue sarcoma. It typically arises in younger to middle-aged adults, while patient age ≥60 years and larger tumor sizes are associated with worse overall and cancer-specific survival. We present the case of a 70-year-old woman with a one-year history of a progressively enlarging, tender erythematous plaque on the left clavicle. Differential diagnosis included dermatofibroma, DFSP, leiomyoma, cutaneous leiomyosarcoma, cutaneous lymphoma, and leukemia cutis. Punch biopsy revealed a CD34-positive spindle cell neoplasm, and fluorescence in situ hybridization confirmed COL1A1 rearrangement, consistent with DFSP. The patient underwent Mohs micrographic surgery, with clear margins achieved after two stages. This case highlights an atypical age of presentation and reinforces the importance of early biopsy and molecular confirmation for accurate diagnosis. Mohs micrographic surgery remains the preferred treatment, offering precise tumor margin control and minimizing recurrence risk compared to wide local excision, while systemic options such as imatinib are reserved for unresectable or metastatic disease. Early recognition and intervention are especially critical in older patients, given their higher risk of adverse outcomes.

## Introduction

Dermatofibrosarcoma protuberans (DFSP) is a rare, low-grade cutaneous soft tissue sarcoma, originating from dermal fibroblasts, that accounts for approximately 1-2% of all soft tissue sarcomas, with an annual incidence rate of 6.25 cases per million person-years in the United States [1,2]. This locally aggressive tumor is characterized by distinctive molecular alterations, most commonly involving the COL1A1-PDGFB gene fusion resulting from chromosomal translocation t(17;22)(q22;q13) or supernumerary ring chromosomes, which leads to constitutive upregulation of platelet-derived growth factor beta (PDGFB) expression and continuous autocrine activation of platelet-derived growth factor receptor (PDGFR) B [3].

The pathogenesis of DFSP involves aberrant PDGFR signaling, making it amenable to targeted therapy with imatinib mesylate, a tyrosine kinase inhibitor that blocks PDGFR activation [4]. However, complete surgical resection with negative margins remains the gold standard treatment and primary prognostic factor, with Mohs micrographic surgery preferred for its superior margin control and tissue conservation compared to wide local excision, due to the high rate of local recurrence [5].

DFSP presents a significant diagnostic challenge due to its non-specific clinical appearance and propensity for misdiagnosis. The tumor typically manifests as an indurated, flesh-colored or erythematous plaque that may be mistaken for benign conditions such as keloids, hypertrophic scars, or lipomas, leading to delayed diagnosis and increased morbidity [6]. Early clinical symptoms are often non-specific, and the condition can remain unrecognized for extended periods, contributing to a high incidence of misdiagnosis.

Recent epidemiological analysis using the Surveillance, Epidemiology, and End Results (SEER) database noted a 2-5% risk of metastasis, and indicated that older age (≥60 years) and larger tumor size (≥3 cm) negatively impacted overall survival and cancer-specific survival, underscoring the importance of early diagnosis and effective surgical intervention [7]. The present case exemplifies the clinical challenges in diagnosing and managing DFSP in an elderly patient and demonstrates the efficacy of Mohs micrographic surgery in achieving complete tumor excision with minimal tissue loss.

## Case presentation

**Figure 1:**
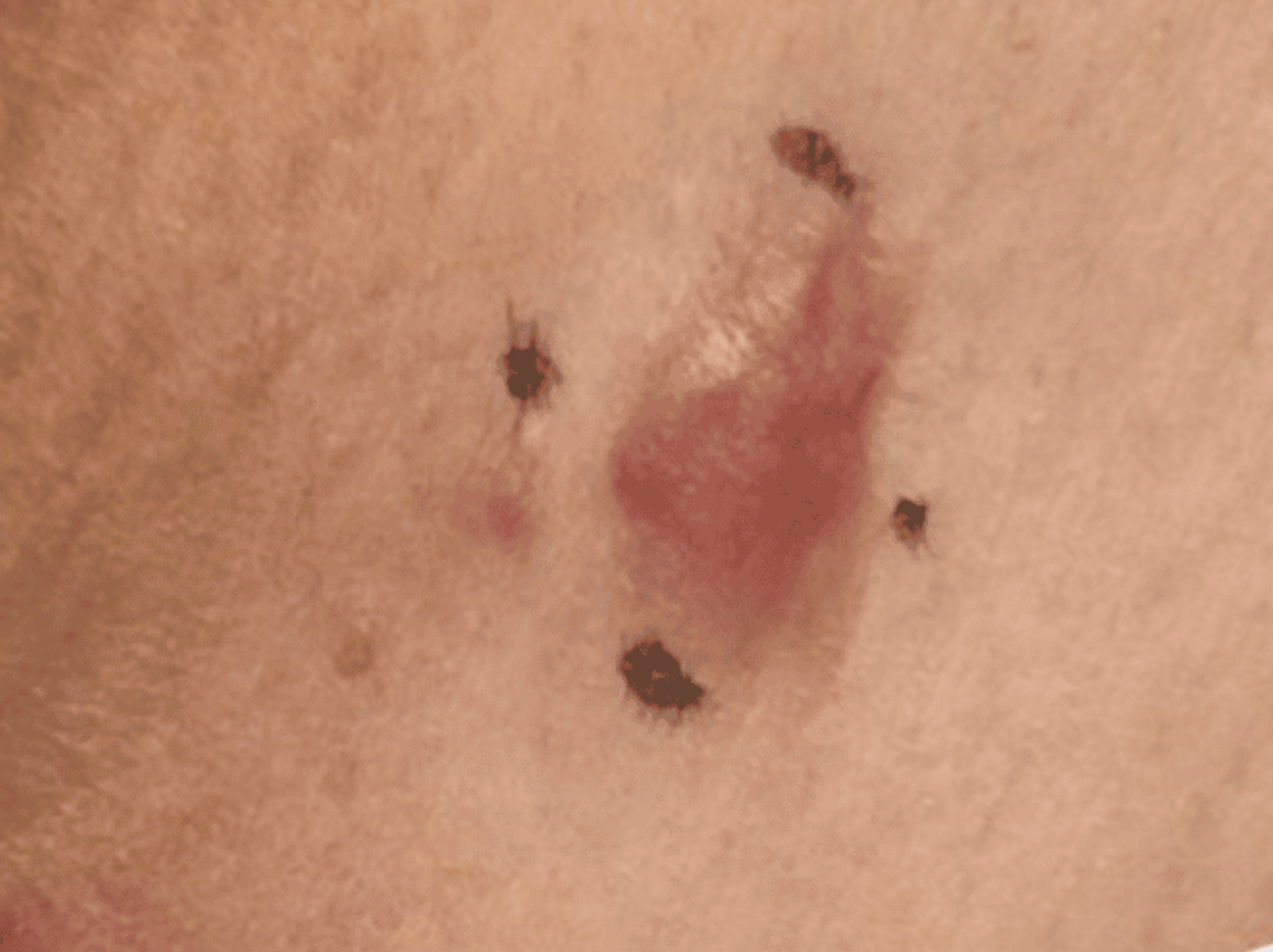
Well-defined, erythematous nodular plaque measuring approximately 2.0 × 1.5 cm overlying the left clavicle of the patient The lesion had been progressively enlarging over one year and was notably tender on palpation.

A 70-year-old female patient, with a history of hypertension, atrial fibrillation, and hyperlipidemia, presented for the evaluation of a one-year progressively enlarging, tender lesion on the skin overlying her left clavicle (Figure 1).

She denied any preceding trauma and personal or family history of skin cancer.

**Figure 2:**
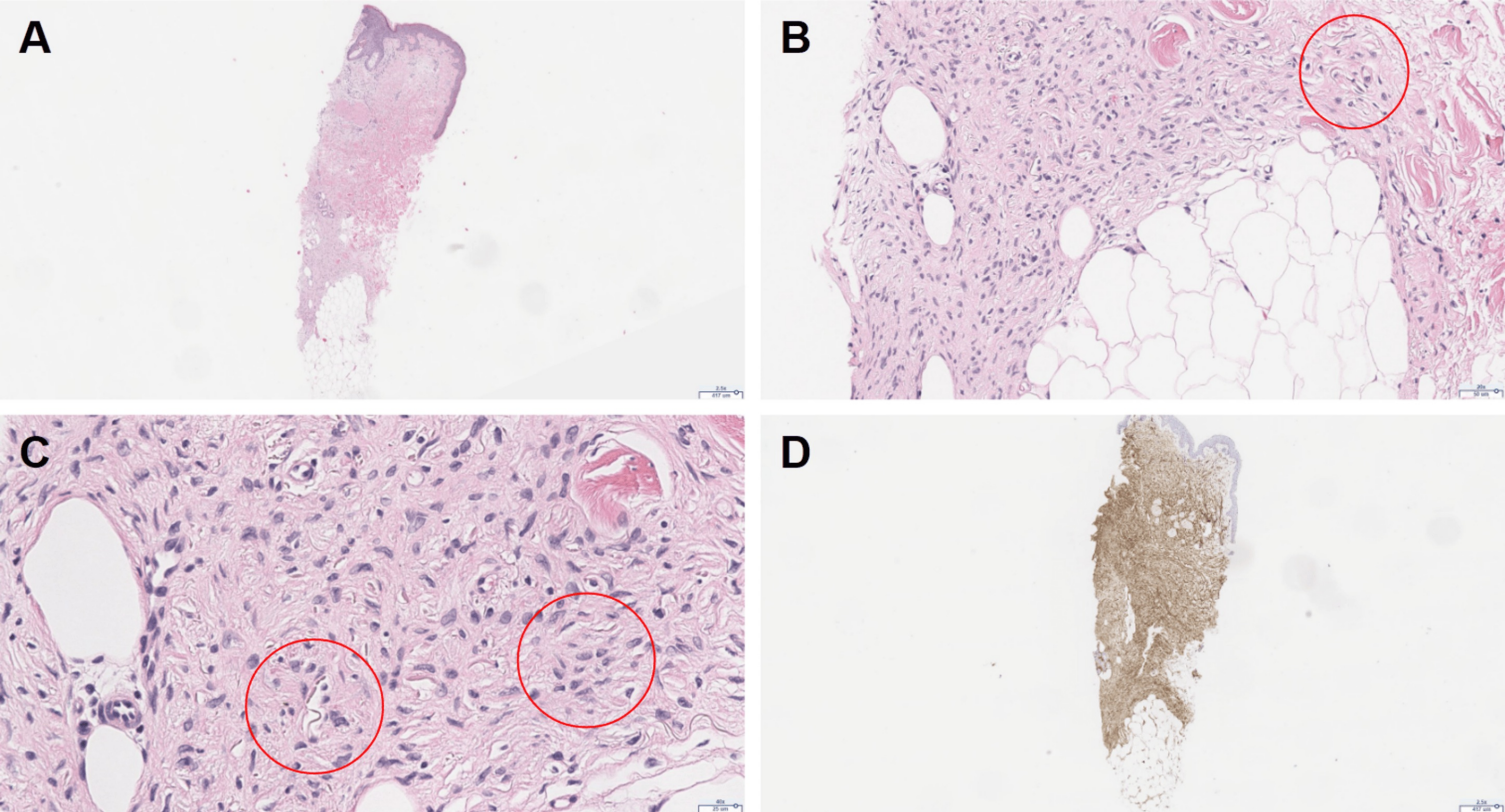
Immunohistochemical staining A: 3-mm punch biopsy from the clavicular lesion stained with hematoxylin and eosin at low power 2.5x. Scale bar, 417µm; B-C: Neoplastic spindle cells present in the dermis and extending into the subcutis. Scale bar, 50µm-25µm; D: Biopsy with CD34 immunohistochemical staining showing positive staining in the neoplastic spindle cells.

Physical examination revealed a well-defined erythematous nodular plaque measuring 2 cm by 1.5 cm on the left clavicular skin. It was significantly tender on palpation. Differential diagnosis was broad, including dermatofibroma, DFSP, leiomyoma, cutaneous leiomyosarcoma, cutaneous lymphoma, and leukemia cutis. A 3 mm punch biopsy was performed, with pathology showing a CD34-positive spindle cell neoplasm extending to biopsy margins, consistent with DFSP (Figure 2).

Molecular confirmation was obtained through fluorescent in-situ hybridization (FISH) analysis, which detected an unbalanced COL1A1 rearrangement consistent with the presence of ring or marker chromosomes containing multiple copies of the rearranged genomic material.

The patient subsequently underwent Mohs micrographic surgery. In the first stage, two tissue sections were processed according to Mohs histological technique and examined microscopically. Areas of residual tumor were identified and marked on the reference map. During the second stage, additional tissue was excised from areas showing residual tumor. One tissue section was processed and examined, revealing tumor-free margins, indicating complete excision of the neoplasm. Surgical margins were tumor-free after two stages of the Mohs surgery.

The patient tolerated both stages of Mohs surgery without complications, and the wound was healing well at the one-month follow-up. She was advised to return for evaluation if new nodules, plaques, firm areas within the scar, or non-healing or enlarging lesions developed at the excision site. At nine months post-procedure, no recurrence has been observed.

## Discussion

This case exemplifies several important clinical and pathological features of DFSP, while highlighting unique aspects that warrant discussion. In an analysis of the Surveillance, Epidemiology, and End Results (SEER) registry of 7748 DFSP patients from 2000-2018 by Maghfour et al. [7], the peak incidence of DFSP was among individuals aged 30-49 years, making our patient's diagnosis at 70 years notably atypical. Epidemiological data indicate that age greater than 60 years is associated with significantly worse overall and cancer-specific survival, emphasizing the clinical significance of timely diagnosis and intervention in older adults.

Our patient’s lesion displayed classical clinical features of DFSP, including progressive growth, erythema, and tenderness. The diagnostic approach followed established protocols with an initial punch biopsy revealing the characteristic histological pattern of uniform spindle cells. The immunohistochemical profile showed CD34 positivity with S100 and SOX10 negativity, which is typical for DFSP and helps differentiate it from other spindle cell neoplasms such as nerve sheath tumors [8]. In addition, molecular confirmation via COL1A1 rearrangement cinched the diagnosis of DFSP [3].

The therapeutic efficacy of Mohs micrographic surgery for DFSP management, as demonstrated in this case, aligns with established guidelines advocating its use for precise tumor margin control, significantly minimizing recurrence risk compared to wide local excision [5]. 

For unresectable or metastatic DFSP, imatinib mesylate represents an important therapeutic option. This tyrosine kinase inhibitor targets the aberrant PDGFR signaling pathway characteristic of DFSP, with studies showing clinical response rates of 55-60% [9]. While not required in this case due to the successful surgical treatment, imatinib may be considered for neoadjuvant therapy in cases where complete surgical excision could result in significant functional or cosmetic impairment. 

Long-term follow-up remains crucial for DFSP patients, as local recurrence can occur even years after apparently complete excision. Mohs surgery has dramatically improved these outcomes [10], but continued monitoring is essential to detect any signs of recurrence. 

## Conclusions

This case highlights the value of maintaining clinical suspicion for DFSP, particularly in older adults presenting with slowly progressive, symptomatic dermal lesions. In such contexts, early biopsy and histopathologic evaluation can help establish a timely diagnosis and guide management to optimize clinical outcomes. Recognizing atypical presentations outside the more common age range may help clinicians consider DFSP in their differential diagnosis and avoid delayed treatment.

Although surgical excision with margin control typically yields favorable outcomes, DFSP carries a persistent risk of local recurrence, emphasizing the need for careful, long-term follow-up. Continued reporting of uncommon presentations can enhance awareness of DFSP’s varied clinical spectrum and support a more nuanced approach to diagnosis and management.
